# Feeding in Forest Chimpanzees: Do Food Type and Canopy Location Predict Positional Behavior?

**DOI:** 10.1002/ajpa.70204

**Published:** 2026-02-22

**Authors:** Laura MacLatchy, Sharifah Namaganda, Lauren Sarringhaus

**Affiliations:** ^1^ Department of Anthropology University of Michigan Ann Arbor Michigan USA; ^2^ University of Michigan Museum of Paleontology Ann Arbor Michigan USA; ^3^ Department of Zoology, Entomology and Fisheries Sciences Makerere University Kampala Uganda; ^4^ Department of Biology James Madison University Harrisonburg Virginia USA

**Keywords:** canopy, chimpanzee, fruit, leaves, positional behavior

## Abstract

**Objectives:**

Distinctive aspects of great ape anatomy and positional behavior, including upright torso stability and suspension, are hypothesized to have evolved to facilitate ripe fruit‐eating in the terminal zone of tree canopies at large body size. Fossil discoveries challenge this view, and we test this perspective by investigating relationships among feeding posture, food type, and canopy zone in chimpanzees.

**Materials and Methods:**

Focal follow data were collected over 10 months on 103 chimpanzees from Ngogo, Kibale Forest, Uganda. Generalized Linear Mixed Modeling was used to examine the effects of food, canopy location, and age on arboreal feeding posture versatility. Branch size and number, and tree size and species were also examined.

**Results:**

When considering all trees, consuming young leaves versus ripe fruit positively affected versatility. When examining only large‐canopy trees, eating leaves versus ripe fruit had no effect, but unripe versus ripe fruit had a negative effect. Terminal zone feeding was associated with an increase in versatility, and in this zone, consuming leaves versus ripe fruit had a negative effect. Versatility was inversely correlated with age, varied across tree species, and was higher on small branches and in small trees.

**Discussion:**

A positive “terminal zone effect” on versatility was detected in large trees, with behavioral impact varying by food type. Within the forest as a whole, consuming leaves over ripe fruit increased versatility. Thus, food type and availability may, in different combinations, lead to equifinality in great ape positional versatility. Hominoid morphofunctional specializations could thus evolve in many ecological contexts.

## Highlights

- Ngogo chimpanzees are most suspensory when eating leaves.
- But while in the terminal canopy, positional behavioral versatility is higher when eating fruit.
- Great ape behavioral specializations may have evolved via multiple ecological paths.

## Graphical abstract

Ngogo chimpanzees from Kibale National Park, Uganda use versatile postures, like suspension, more when eating leaves than other foods. However, in the terminal canopy, eating fruit is most associated with versatility. Food type and availability, in different combinations, produce similar behaviors. Hominoid morphofunctional specializations could thus evolve in diverse ecologies.

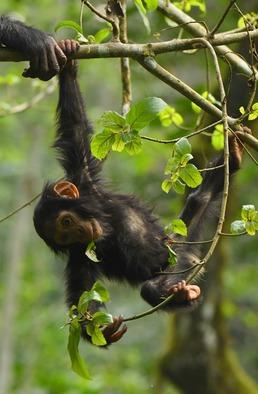

## 1 Introduction

Elucidating the links among anatomy, behavior and arboreality is foundational to the study of primate positional behavioral ecology, or how a primate's movements and body positions relate to its habitat. A well‐known example is Matt Cartmill's visual predation hypothesis (Cartmill 1974), which elegantly proposed that grasping, the most significant of all basal primate adaptations, evolved not solely for arboreality, but primarily for navigating small branches in search of prey under low light conditions. Along with Katherine Milton, Cartmill also explored how large body size impacted arboreality, noting that great apes must “…either utilize disproportionately large supports or distribute weight over several supports at once.” (Cartmill and Milton 1977, 265) Drawing on the work of earlier scholars (e.g., Avis 1962; Grand 1972; Fleagle 1976), these authors described a suite of anatomical features related to large‐bodied hominoid arboreality. These included traits associated with stiffening the spine to maintain an orthograde posture, broadening of the thorax with reorientation of the scapula, increased length and joint mobility of the forelimbs and obviation of the tail. Collectively, these anatomical features help define the hominoids and are well‐established as underlying hominoid positional behavioral versatility.

The term versatility can be used as a general but illustrative descriptor referring to the ability of anatomical complexes to lend themselves to a range of functions (e.g., Larson et al. 2000; Kivell 2016). Recently, it was introduced as a specific term to describe a set of large‐bodied hominoid positional behavior patterns facilitated by two morphofunctional complexes (MacLatchy et al. 2023). First, the trunk is capable of sustained dorsostability due to features such as shortening of the lumbar spine (in terms of the craniocaudal length and number of vertebral bodies (e.g., Keith 1923; Schultz 1961; Rose 1975)) and more dorsally located lumbar vertebral transverse processes (e.g., Shapiro 1993; Ward 1993; Sanders and Bodenbender 1994). Second, there is high mobility of the limb joints, particularly the hip and shoulder (e.g., Ruff 1988; MacLatchy and Bossert 1996; Hammond 2014; e.g., Larson 1993; MacLatchy et al. 2000; Arias‐Martorell 2019). In conjunction with an erect trunk, such mobility enables the forelimbs and hindlimbs to move freely in different directions rather than be restricted to primarily parasagittal planes below a more horizontally‐oriented trunk (Hunt 2016). Although application of the term versatility was largely to locomotor behaviors (MacLatchy et al. 2023), we here extend it to postural behaviors.

**FIGURE 1:**
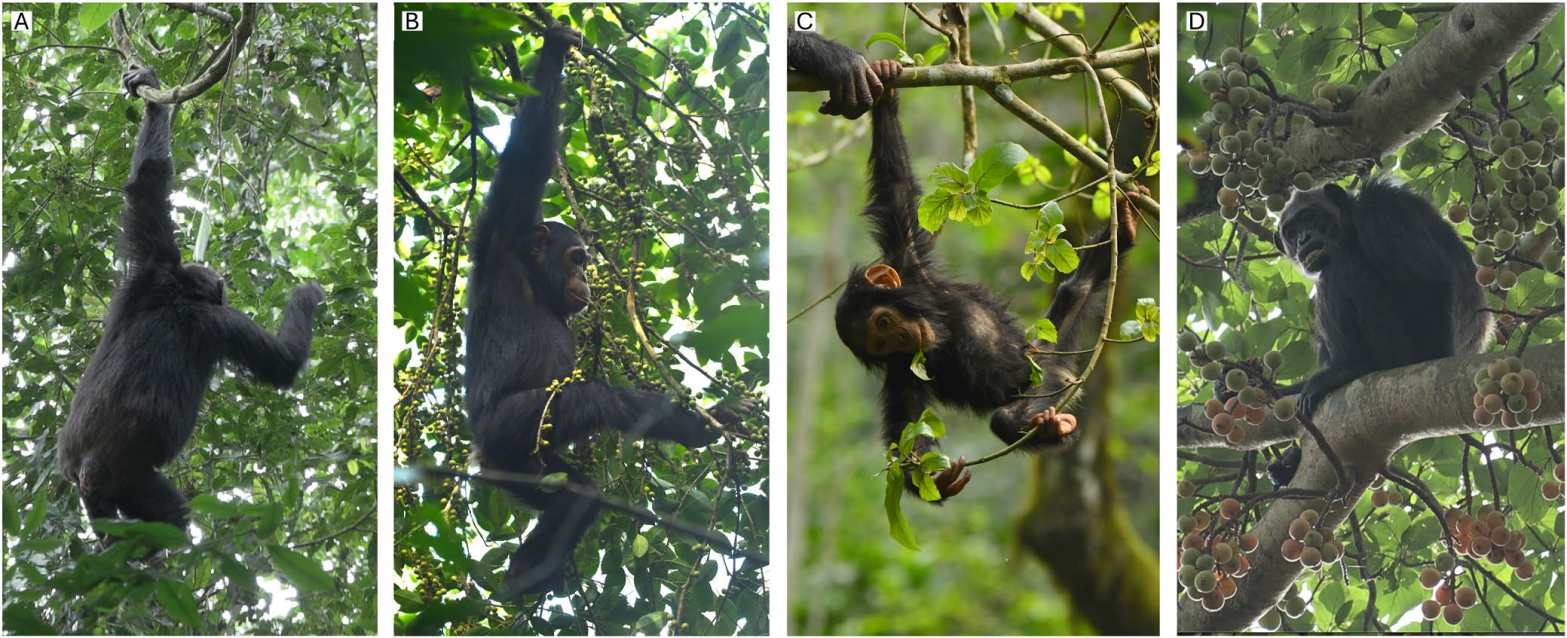
Examples of postures in chimpanzees. Versatile postures: (A) Orthograde forelimb suspending on a small support while feeding on leaves; (B) Orthograde forelimb suspending on a small support while feeding on fruit. Orthograde suspending requires both lower back stability and high limb mobility; (C) Forelimb‐hindlimb suspending on small supports while feeding on leaves. This posture requires high limb mobility and is a rare form of suspension among chimpanzees. Non‐versatile posture: (D) Sitting on a large support while feeding on fruit. This posture is the most common exhibited during feeding and does not require the morphofunctional specializations discussed in the text. See Table S1 for definitions of postures and Methods for definitions of support sizes.

Grouping behaviors under the term “versatility” serves an important purpose when translating suites of anatomical features to capacities for positional behaviors (Figure 1). For example, features permitting high shoulder mobility, such as a wide scapular glenoid fossa (e.g., MacLatchy et al. 2000; Fannin et al. 2023) and a large, spherical humeral head elevated above the level of the greater tubercle (e.g., Larson 1993), enable hominoids to adopt postures such as forelimb suspension and postural bridge, as well as perform movements such as extended‐elbow vertical climbing and brachiation. Such translation is critical for postcranial functional reconstruction in paleontology. As this paper aims to bridge the disciplines of positional behavioral ecology and positional behavioral reconstruction, the term *versatility* will be used consistently throughout.

Versatile behaviors make up approximately half or more of arboreal movements among ape species for whom arboreal locomotion has been similarly documented but play a more limited role in arboreal postural behaviors (Nowak and Reichard 2016a). In this study of postural behaviors, the term versatility will refer to four positional behavioral categories: suspension, postural bridge, orthograde stand, and cling (Tables 1 and S1). Torso stability while upright (the first morphofunctional complex) is critical for versatile postures in large‐bodied hominoids, as once the fore‐ and hindlimbs are freed from the constraints of a habitually pronograde torso, they may be deployed in different directions (facilitated by the second morphofunctional complex) allowing an animal to distribute body weight by concurrently grasping multiple supports and so more fully explore a tree crown. All four postural categories involve orthogrady and/or high joint mobility facilitating limb movements outside parasagittal planes. Suspension in chimpanzees is typically orthograde, though rare exceptions exist, such as forms involving the hindlimb; however, these still involve significant limb mobility (Figure 1). As for the other postures we consider, postural bridging always requires high limb mobility, and in chimpanzees sometimes involves orthogrady or semi‐orthogrady. Both orthograde standing and clinging are by definition orthograde.

**TABLE 1:** Observational focal follow variables.

Variables	Categories	
Age and sex	Infant (*N* = 494) 0.1–5 years, *N* = 17, 9F, 8M	
	Juvenile (*N* = 1540) 5.1–10 years, *N* = 20, 10F, 10M	
	Adolescent (*N* = 1585) 10.1–15 years, *N* = 18, 2F, 16M	
	Adult female (*N* = 1994) > 15 years, *N* = 25	
	Adult male (*N* = 2367) > 15 years, *N* = 23	
Postural mode^a^	Versatile (*N* = 419)	Nonversatile (*N* = 7561)
	Cling	Lie
	Forelimb hindlimb suspend	Pronograde stand
	Hindlimb suspend	Sit
	Orthograde forelimb suspend	Squat
	Orthograde quadrumanous suspend	
	Orthograde stand	
	Postural bridge	
	Pronograde suspend	
Food type	Ripe fruit (*N* = 4586)	
	Unripe fruit (*N* = 1134)	
	Young leaves (*N* = 1161)	
	Mature leaves^b^ (*N* = 17)	
	Flowers (*N* = 685)	
	Other^b^, ^c^ (*N* = 397) pith, bark, roots, seeds, meat, wood, dirt	
Tree DBH^d^	< 10 cm (*N* = 424)	
	10–< 20 cm (*N* = 351)	
	20–< 40 cm (*N* = 626)	
	≥ 40 cm (*N* = 6109)	

*Note:* The second values are numbers of different individuals sampled for each sex.

Abbreviations: F, female; M, Male.

^a^ Postural Mode definitions from (Hunt et al. 1996; Thorpe and Crompton 2006; Sarringhaus et al. 2014; Sarringhaus et al. 2024; See Supporting Information S1).

^b^ Indicates excluded from Generalized Mixed Models (GLMM).

^c^ The “others” category comprised a heterogeneous mix of items of arboreally harvested foods and terrestrial items that were subsequently consumed in trees. We excluded the other foods category from further analysis, as these items fall outside the scope of our hypotheses. Likewise, mature leaves were omitted from GLMM analysis due to their rarity in the dataset (only 17 of 7980 total feeding observations).

^d^ Tree diameter at breast height was not available for 56 observations. Mature leaves and foods in the others category are excluded from these counts.

To date, it has generally been expected that insights related to the emergence, evolution and significance of derived hominoid postcranial traits and their behavioral correlates would come primarily from two sources: the discovery of fossils of stem and crown taxa; and detailed anatomical, developmental and genetic study of the characters under scrutiny. While these approaches remain essential, further insight can be gained through empirical investigations into how versatile positional behaviors contribute to the success of living great apes. By studying wild apes and documenting the ecological contexts in which versatile behaviors are utilized, researchers can generate and test hypotheses about the functional significance of postcranial traits and their impact on fitness. Such an approach can provide a deeper understanding of why specific traits evolved and how they may have contributed to the diversification and evolution of hominoids.

The central tenet of hypotheses advanced to explain the adaptive significance of versatile behaviors in hominoids is that these behaviors enhance foraging performance. Specifically, they enable medium and large bodied primates to access high quality ripe fruit located in areas of the tree crown with small, flexible supports, including the upper and most notably the terminal canopy (e.g., Avis 1962; Napier 1967; Cartmill and Milton 1977; Temerin and Cant 1983; Cachel 1979; Cant 1987a). Suspension has been emphasized in most scenarios of hominoid evolution and historically has been viewed as the *sine qua non* expression of derived aspects of hominoid anatomy (e.g., Tuttle 1975; Cachel 1979; Temerin and Cant 1983). Other behaviors such as quadrumanous climbing (Fleagle 1976), vertical climbing (Stern Jr and Susman 1981; Isler 2005), and orthogrady more broadly (Thorpe and Crompton 2006; Nowak and Reichard 2016a) have also been identified as key components of hominoid positional repertoires.

These terminal canopy zone–frugivory hypotheses that address the origins of positional behavioral versatility have relied heavily on the ecological characteristics of extant apes.

As a clade, living apes tend to be forest‐dwelling, with ripe fruit feeding preferences (Rogers et al. 2004; Watts 2012). Ecological exceptions such as mountain gorillas and woodland/savanna chimpanzees are sometimes viewed as inhabiting marginal environments (Remis 1998; Watts 1998; Marchant et al. 2020; Wessling et al. 2020), likely distinct from those inhabited by ancestral forms and so potentially less relevant for reconstructing hominoid evolution.

In this study, we consider the implications of feeding behavioral versatility for the evolution of the large‐bodied arboreal hominoids. Modern arboreal great apes weigh more than 30 kg, with male orangutans and female gorillas far surpassing this threshold (Smith and Jungers 1997). (Male gorillas are less arboreal by comparison.) Consequently, they experience particularly significant challenges navigating the tapering branches of the terminal canopy zone. They also exhibit distinctive anatomical features thought to be associated with these challenges. As noted above, only great apes possess a lumbar column with fewer vertebrae compared to other hominoids, which reduces the distance between the iliac blades and the lower thorax and facilitates lumbar stiffness. The modal number of lumbar vertebrae is five in both hylobatids and humans, but four for members of *Pongo, Pan*, and *Gorilla*, although a small sample of *Gorilla berengei* exhibited a modal number of 3 (Pilbeam 2004).

### 1.1 The Terminal Canopy Zone

We operationalize “terminal canopy zone” as the outer 1/3 of a tree crown. Here, branches are consistently smaller, thinner, younger, and more flexible than branches closer to the center of a tree (e.g., Bertram 1989). Due to increasing hydraulic resistance with tree height (Petit et al. 2008), branches must taper as their length increases to ensure consistent xylem flow to all leaves (West et al. 1999). As a result, trees exhibit a dendritic pattern in which the trunk possesses the thickest diameter, and stem diameter decreases with distance from the central axis.

The phenomenon of tree stems and branches narrowing in diameter with increasing height or at successive forks has been intensively studied (e.g., Salekin et al. 2021). Mammalogists have also explored how animals adapt morphologically and functionally to tree branch attenuation. Grand was among the first to articulate the inherent problem of accessing the terminal zone: “Harvesting from terminal branches is difficult for [other] nonvolant mammals, because trees naturally become more slender in the periphery” (Grand 1972, 198). He observed that as body weight increases, so does the need for specialized morphological adaptations.

### 1.2 Paleontological Evidence for Positional Behavior Versatility in Early Hominoids

Because extant great apes exhibit a preference for ripe fruit, their arboreal positional behavior when eating other foods remains understudied. Furthermore, the focus on ripe fruit in generating hypotheses about hominoid origins is too narrow in light of paleontological evidence from Early Miocene fossil sites in eastern Africa which indicate that early apes utilized a broad range of habitats and foods (Andrews 2015; MacLatchy et al. 2023).

It has been difficult to test whether there was an association between versatile behaviors, fruit eating and forest‐dwelling in the earliest apes. The estimated age for the hominoid‐cercopithecoid divergence is ≥ 25 Ma (Steiper and Seiffert 2012), yet few fossil sites straddle this critical period (Stevens et al. 2013). Moreover, it is rare to recover both craniodental and postcranial fossils in association, and even rarer to find them in a depositional environment amenable to detailed paleoecological reconstruction. However, recent behavioral interpretations of fossils assigned to *Morotopithecus bishopi*, a ~40 kg, 21 Ma hominoid from Uganda, indicate that the oldest evidence of orthogrady in the ape fossil record occurs alongside a diet with a significant folivorous component (MacLatchy et al. 2019, 2023). Features supporting a more stable lower back include a more dorsal orientation and a more dorsal point of origin of the transverse processes, which arise from robust pedicles rather than from the vertebral body (Sanders and Bodenbender 1994) increasing the leverage for the iliocostalis and longissimus dorsi muscles that function to resist flexion in the lumbar region (Shapiro 1993). Furthermore, the femur of *Morotopithecus* suggests ape‐like limb use: it is short, has a strong mid‐shaft, and features a broad knee joint, all of which would have biomechanically favored climbing at large body size. Folivory is supported by an elongated tooth row, and a second lower molar with relatively well‐developed shearing crests (MacLatchy et al. 2019).

Detailed paleoenvironmental reconstructions have been carried out for the Moroto II locality where the *Morotopithecus* fossils described above were collected. Carbon isotope values from herbivore dental enamel, pedogenic carbonates, paleosol organic matter and plant waxes all point to the presence of C_4_ grass as well as C_3_ plants, as do phytolith assemblages (MacLatchy et al. 2023; Peppe et al. 2023). Additionally, paleosol analyses suggest a sub‐humid environment, with strongly seasonal precipitation (MacLatchy et al. 2023). Viewed in combination, these paleoenvironmental indicators suggest that *Morotopithecus* inhabited seasonal, grassy woodlands lacking a high, continuous canopy. Furthermore, ripe fruit availability would have fluctuated and been less predictable, perhaps leading to an increased dependence on fallback foods.

While several other early Miocene sites have yielded associated hominoid crania and postcrania, only two sites, Moroto II (MacLatchy et al. 2023) and Rusinga R3 (Michel et al. 2014), are currently characterized by high‐resolution paleoecological data sets incorporating faunal, floral, and pedogenic data. At the 18‐million‐year‐old Rusinga site, the ape *Ekembo heseloni,* estimated to resemble baboons in both body size and sexual dimorphism (McNulty et al. 2025), has been reconstructed as a generalized frugivore (Andrews and Martin 1991). This species inhabited a more forested environment compared to what has been inferred at Moroto II (Michel et al. 2014; Peppe et al. 2023) and is interpreted as a pronograde quadruped (Ward 2015). These findings suggest we are only beginning to uncover the complex associations between diet, habitat, and positional behavior in the evolutionary past of hominoids. Neither of the morphological/paleoecological reconstructions described above for *Morotopithecus* and *Ekembo* conform to expectations based on living apes. The postcranially derived, folivorous ape *Morotopithecus* lived in a seasonal woodland, while the postcranially more primitive and more frugivorous ape *Ekembo* lived in a forest.

Given Miocene fossil evidence revealing the importance of folivory in one of the oldest hominoids, it is relevant to compare hominoid positional behavioral versatility across different feeding contexts, namely, when consuming ripe fruit, unripe fruit, and leaves, to investigate the importance of fruit and terminal canopy usage in ape positional behavioral ecology.

### 1.3 Great Ape Arboreal Positional Behavior in the Wild

Studies of hominoid arboreal postural behavior have emphasized the adaptive significance of versatile postures in chimpanzees (often manual suspension (Hunt 1991, 1992a, 1992b)) and in hominoids more broadly (see review in Hunt 2016). The most common arboreal posture in well‐studied extant hominoids and cercopithecoids is sit/squat (Nowak and Reichard 2016a). Sitting provides stability, as the center of mass tends to be positioned closer to the substrate (e.g., compared to standing), and a relatively greater surface area may come into contact with supports compared to postures that are limited to contact with the hands and/or feet. Beyond stability, sitting is also energetically conservative (Gao et al. 2017). While apes are sitting, they are able to pull branches toward themselves using their relatively long arms (apes have higher intermembral indices than all other anthropoids except spider and woolly spider monkeys (Erikson 1963; Jungers 1985)), which helps to optimize their use of this posture.

However, while monkeys in the trees typically quadrupedal stand with a pronograde torso if not sitting (McGraw 1998; Nowak and Reichard 2016a), chimpanzees have been described as manually suspending with an orthograde torso if not sitting (Hunt 1992a). The picture becomes more complex if other great apes are included (Cant 1987a, 1987b; Melissa Remis 1995; Remis 1998, 1999; Thorpe and Crompton 2006). Nonetheless, torso‐orthograde forelimb suspension remains of considerable adaptive interest, particularly in relation to ecological factors. Hunt (1992a) observed that chimpanzees preferentially engaged in versatile postures (arm suspension), but not locomotion, while in terminal branches and also reported that these branches were primarily used in the context of feeding. Orangutans similarly feed in the terminal zone, utilizing predominantly suspensory behaviors (Myatt and Thorpe 2011).

The role of food type, particularly the contrast between fruit and leaf consumption in shaping hominoid positional behavior, was first explored by Chivers (1974) and Fleagle (1976), who found that the consumption of new leaves by siamangs was more commonly done from sitting positions, while feeding on fruit was more often associated with suspensory positions. The gibbon *Nomascus nasutus* has also been found to be more suspensory when feeding on fruit compared to leaves (Fei et al. 2015). Hunt (1992a) found that the large majority (> 90%) of all arm‐hanging postures practiced by chimpanzees at both Mahale and Gombe, Tanzania occurred during feeding on fruit. However, this pattern was not exclusive to suspension, as all postural categories observed during feeding had the highest frequency during fruit consumption, reflecting chimpanzees' predominantly frugivorous diet (see table 12 in Hunt 1992a). Any association between versatile postures and leaf‐eating in chimpanzees therefore remains unclear.

The quality and location in the canopy of fruit and leaves are key ecological factors influencing primate feeding behavior and preferences. At Kibale National Park, Houle et al. (2007) documented a pattern of vertical stratification in several fruiting trees, with the upper canopy (constituting a substantial portion of the terminal zone) producing larger and more abundant fruit with higher pulp and water content than fruit found in the lower canopy. Young leaves are generally preferred by primates as they have a higher ratio of protein to fiber (Milton 1979; Lambert and Rothman 2015). Nutritional studies at Kibale have shown that the young leaves consumed by chimpanzees (Uwimbabazi et al. 2019) and colobines (Chapman and Chapman 2002; Chapman et al. 2004) are high in protein. The latter two studies also reported that young leaves contain relatively less fiber and are more digestible than mature leaves.

In addition to food type, demographic variables also correlate with positional behavior under certain circumstances, with developmental transitions and sex differences in postural behavior varying across great apes. Thus far, in orangutans, age‐sex classes have been found to exert a marginal influence on support use and positional behavior (Thorpe and Crompton 2005; Myatt and Thorpe 2011). Eastern (mountain) gorillas, by contrast, exhibit significant age differences but adult‐like positional behavior develops as early as age two (Doran 1997). In more frugivorous and arboreal western gorillas, females spend more time arboreally compared to males (Remis 1995; Remis 1998, 1999), and when arboreal, adult female western gorillas exhibit a higher frequency of both orthograde stand and suspensory postures compared to adult males (Remis 1995, 1998). Chimpanzees do not exhibit adult positional repertoires until adolescence (Sarringhaus et al. 2014), making age an important variable to consider in this taxon. While the degree of arboreality is higher in female than male chimpanzees across chimpanzee populations (Doran and Hunt 1994; Doran 1993; Drummond‐Clarke et al. 2024), differences in arboreal postural behavior repertoires between the sexes are minor (Doran and Hunt 1994; Doran 1993; Sarringhaus et al. 2014).

In summary, the ways in which food type, canopy location, and demographic variables influence the execution of great ape positional behavior in the wild remain important to explore. Few studies have focused in detail on feeding posture—the behavior expressed during the act of feeding itself—as well as canopy zone usage and food type, although exceptions exist, such as Hunt's (1992a) research on chimpanzees.

In the case of hylobatids, even the largest gibbons are significantly smaller than the smallest great apes, which somewhat limits their usefulness for understanding great ape feeding posture ecology, despite sharing features such as an orthograde trunk and high limb mobility (Schultz 1930; Tuttle 1975; Reichard et al. 2016). Among hylobatids, patterns of support use vary by both food type and canopy location. For example, Fei et al. (2015) found that *N. nasutus* was more likely to consume fruit in a suspensory posture than when eating leaves. Nowak and Reichard (2016b) observed that *Hylobates lar* primarily sat in the core of the tree, but at the periphery, individuals were almost equally likely to sit (54%) as to suspend (43%), although they did not break down canopy usage by food type. Gittins (1983) reported that *Hylobates agilis* consumed leaves throughout the vertical canopy, while fruit was mainly eaten in the middle canopy. Additionally, *H. lar* was found to primarily use twigs (< 2 cm) when hanging but used twigs, branches (2–10 cm), and boughs (> 10 cm) when sitting (Nowak and Reichard 2016b). However, this pattern may be context dependent as *H. agilis* exhibited this pattern for suspension while feeding but used branches and twigs equally when suspending to forage (Fleagle 1980; Gittins 1983). When considering suspensory substrate usage by food type, Fei et al. (2015) found that twigs and lianas (both small) were more frequently used compared to larger branches and boughs for both fruit and leaves in *N. nasutus* . Further research is required to determine if these findings are generalizable across gibbon species and to clarify how posture in the terminal zone relates to food type.

The relationships among food type, feeding location, and posture have also been explored in non‐hominoid anthropoids (Garber 1992, 1998, 2019; McGraw 1998; Dunbar and Badam 2000; Laird et al. 2022; Lu et al. 2024). While no other primates express the great ape complement of large body size and pronounced shortening and stabilization of the lumbar region, some platyrrhines and colobines have evolved morphofunctional complexes associated with high limb mobility and versatile postures, notably suspension. For example, the wide scapular glenoid fossa and large, spherical humeral head noted above also occur in *Ateles* (Larson 1998; MacLatchy et al. 2000). *Ateles* and other atelines adopt suspensory postures at rates ranging from 33% to 53% (Mittermeier and Fleagle 1976; Mittermeier 1978; Bergeson 1998; Youlatos 2002, 2008), although frequent use of a prehensile tail (e.g., Mittermeier and Fleagle 1976; Johnson and Shapiro 1998) complicates direct comparisons with hominoids. These studies do not find a simple link between food type and suspensory postures. For example, *Ateles paniscus* was found to have similar postural frequencies when feeding on fruit or young leaves (Youlatos 2002), while *Alouatta palliata* and *Ateles geoffroyi* were found to suspend more when feeding on leaves versus fruit (Bergeson 1998). Feeding location in the terminal crown also does not clearly vary by food type as primarily omnivorous *Cebus capucinus* , primarily folivorous *A. palliata* , and primarily frugivorous *A. geoffroyi* all use this zone (*ibid*.). Some Asian colobines also use forelimb suspension (Byron and Covert 2004; Wright et al. 2008; Bailey et al. 2020). Postural data for wild *Pygathrix* during foraging are limited, but data from *Rhinopithecus* indicate suspension is more common when feeding on lichen compared to both fruit and leaves (Grueter et al. 2013). Overall, there is clearly high variability among both apes and monkeys in how type and spatial distribution of food and feeding postures are interrelated.

### 1.4 Chimpanzees as a Model Great Ape

Chimpanzees represent an ideal model for investigating relationships among food type, feeding location, and posture in a large‐bodied ape due to their degree of arboreality and sociality. Although orangutans are highly arboreal, their less gregarious social structure (e.g., Van Schaik and Van Hooff 1996) makes it harder to obtain large behavioral samples across individuals, while gorillas are too terrestrial to provide comparable arboreal data (Doran 1997; Melissa Remis 1995; Remis 1998, 1999).

Among extant great apes, chimpanzees are perhaps the best‐studied with respect to positional behavior (e.g., Hunt 1992a, 1992b; Doran 1993; Doran and Hunt 1994; Sarringhaus et al. 2014; Drummond‐Clarke et al. 2024), and their ecology and diet are well characterized at Ngogo, Kibale Forest, Uganda (Potts et al. 2011; Watts et al. 2012a, 2012b), where the study communities for this research project are located. The Ngogo chimpanzees occur at high population density and have relatively low dependence on terrestrial vegetation (Potts et al. 2011), making these individuals fitting subjects for examining the relationship between arboreal food types, feeding location, and postural behavior during feeding.

### 1.5 Goals and Predictions

In this study, we investigate the links among feeding posture, food type, and canopy zone in forest‐dwelling chimpanzees at Ngogo, Kibale National Park, Uganda. Our primary goal is to determine whether versatile feeding postures are associated with the exploitation of specific food types in specific parts of the tree crown. In particular, we ask whether versatile postures are associated with the consumption of ripe fruit rather than with other foods, particularly in the terminal canopy zone where supports are smaller.

However, given the folivorous adaptations reconstructed for the early hominoid *Morotopithecus*, positional behavioral versatility may also be associated with feeding on leaves. This possibility suggests that existing hypotheses, which focus primarily on ripe fruit foraging in the terminal canopy zone, should be broadened when explaining the origins of positional behavioral versatility, and by extension, the postcranial features that underlie these capacities.

Furthermore, our analysis extends to examining whether the canopy zone affects the versatility exhibited by chimpanzees while consuming not just fruit and leaves, but two other major food sources for chimpanzees, unripe fruit and flowers.

As this is a complex topic, we explore whether versatility varies with three key parameters: relative support size, the number of weight‐bearing supports and tree size. We predict that when using single supports, smaller supports will be associated with higher versatility than larger supports. We also consider the impact of the simultaneous use of multiple weight‐bearing supports, hypothesizing that versatility will be greatest when feeding involves weight distribution across multiple small supports, rather than across multiple larger supports. We use diameter at breast height (DBH) as a proxy for tree size. Because larger trees typically bear larger branches, we expect feeding in larger trees to involve larger supports and so predict lower overall postural versatility in such trees.

In this study we also examine age as a variable due to its strong correlation with body size. In addition, selective pressures may act differently on different developmental stages independent of body size (Carrier 1996; Irschick et al. 2000; Young and Shapiro 2018; Bezanson 2009), further strengthening the rationale to examine age. At Ngogo, juveniles and infants have been shown to be more suspensory than adults and adolescents (Sarringhaus et al. 2014); thus, we expect these younger age classes to exhibit higher versatility specifically when feeding. Given that sex‐based differences in postural behavior are minor in adults and absent in subadults at Ngogo (Sarringhaus et al. 2014), we expect no sex differences in feeding behavior versatility. We nonetheless examine the effect of sex on postural versatility among adults, given that male *Pan troglodytes schweinfurthii* are on the order of 20%–26% larger than females (Uehara and Nishida 1987; Pusey et al. 2005).

## 2 Materials and Methods

### 2.1 Study Site and Study Subjects

The Ngogo study area is in the center of Kibale National Park, Uganda. It lies at 1400 m above sea level and receives 1447 ± 149 mm of annual rainfall (1997–2022; Mitani et al. 2024). The study area primarily contains old‐growth, evergreen forest punctuated by colonizing forests, swamp forests, and grasslands (Struhsaker 1997; Potts et al. 2020) with chimpanzees utilizing predominantly old‐growth forest (Watts et al. 2012a). Our study included individuals from two communities at Ngogo. At the time of data collection, the population comprised approximately 200 individuals occupying a ~35 km^2^ territory (Mitani et al. 2010; Wood et al. 2017).

### 2.2 Focal Follows

Two hour focal follows with two‐minute instantaneous scan samples were conducted on target individuals by SN during 10 months in 2020 and 2021. Focal follows included data collection on individual ID, age, sex, positional behavior, tree zone location, context, food type and food species, resulting in 35,888 observations across 120 individuals. The current study includes all arboreal postural observations during feeding from that data set, which amounted to 7980 observations for 103 chimpanzees. The number of individuals within each age‐sex group was comparable (Table 1). The number of observed feeding bouts per individual ranged from 1 to 10 days, with a mean of 4.56 (SD = 2.57). Sampling was generally balanced across age–sex categories, with the exception of the infant group: 104 bouts were recorded for adult females, 121 for adult males, 110 for adolescents, 111 for juveniles, and 52 for infants.

### 2.3 Age Groups

We combined age and sex into a single categorical variable with the following groups: infants (0.1–5 years), juveniles (5.1–10 years), adolescents (10.1–15 years), adult males (> 15 years), and adult females (> 15 years) (Table 1). A categorical approach to age was preferred in part because these groupings serve as proxies for body size. Since body mass ~ plateaus at adulthood (at approximately 15 years of age), treating age as a continuous variable would obscure size‐related effects. We analyze adult males and females separately given their body size dimorphism. To account for potential age‐related differences in positional behavior versatility, age group was incorporated as a fixed effect in the generalized linear mixed models (GLMMs) (see below).

### 2.4 Posture

The act of feeding as defined in our study involved active chewing and reaching for food and was almost always accomplished in a postural (vs. locomotor) context. We thus follow other scholars in examining arboreal posture to explore feeding (e.g., Fleagle 1976; Hunt 1992a; Remis 1998; Myatt and Thorpe 2011). Postures were categorized based on definitions from the literature (Hunt 1996; Thorpe and Crompton 2006; Sarringhaus et al. 2014; see Table S1). We grouped postures into two categories: non‐versatile and versatile (Table 1). Non‐versatile postures included positions commonly observed in all primates and that do not depend on the postcranial specializations characteristic of hominoids. Versatile postures included suspension, orthograde stand, cling, and postural bridge.

### 2.5 Food

Our food categories during data collection were flowers, unripe fruit, ripe fruit, young leaves, mature leaves, and others (Table 1). As mature leaves comprise an insignificant component of the diet (Table 1), we follow previous studies considering chimpanzee dietary ecology by focusing on young leaves (e.g., Carlson et al. 2013; McLennan 2013; Uwimbabazi et al. 2019), and we exclude mature leaves from our analyses. The “other” category was also excluded as these items fall outside the scope of our hypotheses. All foods were recorded using the species names provided in Watts et al. (2012a), with the exception of the redesignation of *Morus mesozygia* as *Morus lactea*.

### 2.6 Canopy Zone

The canopies of individual feeding trees with a DBH ≥ 20 cm were used in our GLMM analyses incorporating canopy zone as a predictor, as this size threshold has been used to denote large tropical trees that reach the full canopy (e.g., Panzou et al. 2021). Such trees have a complex, spreading crown which was conceptualized as a partial sphere and divided into two zones: the terminal zone, representing the outer one‐third of the canopy, and the non‐terminal zone, or the central two‐thirds of the canopy.

### 2.7 Branch Size and Number

Support diameters were categorized according to the size of an adult chimpanzee's hand, following Bezanson (2009), who argued this method is more consistent and replicable than estimating absolute size of support. A “large” support cannot be encompassed by two adult hands together; a “medium” support can be encompassed by two hands, but not one; and a “small” support can be encompassed by a single hand. The number of weight‐bearing supports was classified as follows: “large and smaller” requires at least one large support and one additional support; “medium and smaller” requires at least one medium support and one other support that is medium or smaller; or “small and smaller”, requiring at least two small supports but none that are larger. Whether a support was weight bearing was inferred by observing the positioning of the torso relative to the rest of the body and the support; the appearance of limbs, hands and feet; and the degree to which a support deformed and rebounded when weight was applied and shifted (Hunt et al. 1996).

### 2.8 Tree Size

Diameter at breast height (DBH) was used to assess tree size and was grouped into four size categories: < 10, 10–< 20, 20–< 40 and ≥ 40 cm.

### 2.9 Data Summarization and Visualization

We examined the relative frequencies of versatile behavioral events by food category and according to branch size, branch number, and DBH. We also considered the percentage of support size usage for each single support size category for each DBH category. For the most commonly consumed tree species, we also document versatility and the proportion of trees with DBH ≥ 20 cm.

### 2.10 Analyses

To determine which variables influence the incidence of postural versatility, we used GLMMs to assess the main effects of food, canopy location, and their interaction, as well as age group, on the incidence of postural versatility. All models were fitted using glmer() from the lme4 package, R(v 4.4.3), assuming a negative binomial distribution with a log link function. In all models, individual ID was included as a random effect to account for repeated observations. An offset term (log‐transformed total number of behavioral records per individual) was included to control for variation in sampling effort across individuals.

We constructed a count‐based dataset in which each row represented a complete feeding bout by a single individual within a single tree. Postural records were collected every 2 min during an up to 2‐h‐long focal follow. For each bout, the total number of versatile postures was calculated as a percentage of all postural observations during that feeding bout. A total of 7980 observations from the focal follows yielded 498 feeding bouts, with each bout representing a single chimpanzee over the course of a maximum of 60 observations. To aid interpretation of Model 3 (described below) examining the interaction between canopy zone and food type, the percentage of time that a chimpanzee fed in the terminal zone was mean‐centered for each bout prior to analysis. This made it possible to interpret the categorical effects of food at the average level of terminal zone use. For all models, ripe fruit and adult males served as the reference categories for food type and age group, respectively.

### 2.11 Model Construction

Our first model considered the main effects of food type and age group on the expression of versatile posture across all arboreal feeding bouts (*N* = 498):

$$\text{Model}\;1:\text{Versatility}\sim \text{Food}+\mathrm{Age}\;\text{Group}+\left(1\;|\;\mathrm{ID}\right)$$

Given that smaller trees may not have a complex canopy, we further considered two models to predict versatility using a data set including only large trees (≥ 20 cm DBH) (*N* = 367 bouts):

$$\text{Model}\;2:\text{Versatility}\sim \text{Canopy zone}+\text{Food}+\mathrm{Age}\;\text{group}+\left(1\;|\;\mathrm{ID}\right)$$

$$\text{Model}\;3:\text{Versatility}\sim \text{Canopy zone}\times \text{Food}+\mathrm{Age}\;\text{group}+\left(1\;|\;\mathrm{ID}\right)$$

Model 2 allowed us to investigate the effects of canopy zone and food type independently, while Model 3 enabled us to also assess whether the influence of canopy zone on versatility varies by food type, an important theoretical consideration for this study.

To further understand the relationship between food type, canopy location and the expression of postural versatility while feeding in large trees, we used the emtrends() function from the emmeans package in R to estimate marginal trends of terminal zone effects for each food type. Pairwise comparisons of these slopes were then conducted to assess the differences between food types. Because the model was fitted on the log scale, contrasts were exponentiated and expressed as percentage differences in predicted versatility to aid interpretation. We also used the emmeans() function to perform pairwise comparisons among the different age groups. For all sets of comparisons, Tukey's adjustment for multiple comparisons was applied to assess statistical significance.

## 3 Results

### 3.1 Descriptive

While feeding arboreally, chimpanzees overwhelmingly used non‐versatile postures (94.75%), principally sitting (90.68%) (Table 2). Versatile postures were used in 5.25% of feeding observations, mostly in suspension (4.09%) (Table 2).

**TABLE 2:** Percentages of arboreal feeding postures.

Postural category	Overall posture %	Overall category %	Overall subcategory %	Versatile category %	Versatile subcategory %
Non‐versatile	94.75				
Sit		90.68			
Squat		2.31			
Lie		0.44			
Pronograde stand		1.33			
Versatile	5.25				
Orthograde stand		0.58		11.05	
Postural bridge		0.06		1.15	
Cling		0.53		10.10	
Suspension		4.09		77.90	
Pronograde			0.18		3.34
Forelimb‐Hindlimb			0.09		1.71
Orthograde Forelimb			3.60		68.57
Orthograde Quadrumanous			0.21		4.00
Hindlimb			0.01		0.20

Of the versatile postures used (Table 2), 93.9% were orthograde (orthograde stand, orthograde forelimb suspend, orthograde quadrumanous suspend and cling), reflecting the central role that a stable torso plays in versatile postures. An additional 2.9% of versatile postures were semi‐orthograde (or approached orthogrady) and required exceptional limb mobility and positioning of the limbs outside parasagittal planes (postural bridge, forelimb‐hindlimb suspension). The remaining 3.6% of versatile postures were non‐orthograde forms of suspension that in chimpanzees involve high limb mobility to be sustained (pronograde suspension and hindlimb suspension).

Ripe fruit accounted for more than half of all feeding observations (57.47%). Young leaves and flowers accounted for a similar proportion of feeding observations (14.55% and 14.21% respectively), while proportions for flowers (8.58%), “other” foods (4.97%) and especially mature leaves (0.21%) amounted to much less. The latter two food types are not included in any of the following visualizations or analyses.

**FIGURE 2:**
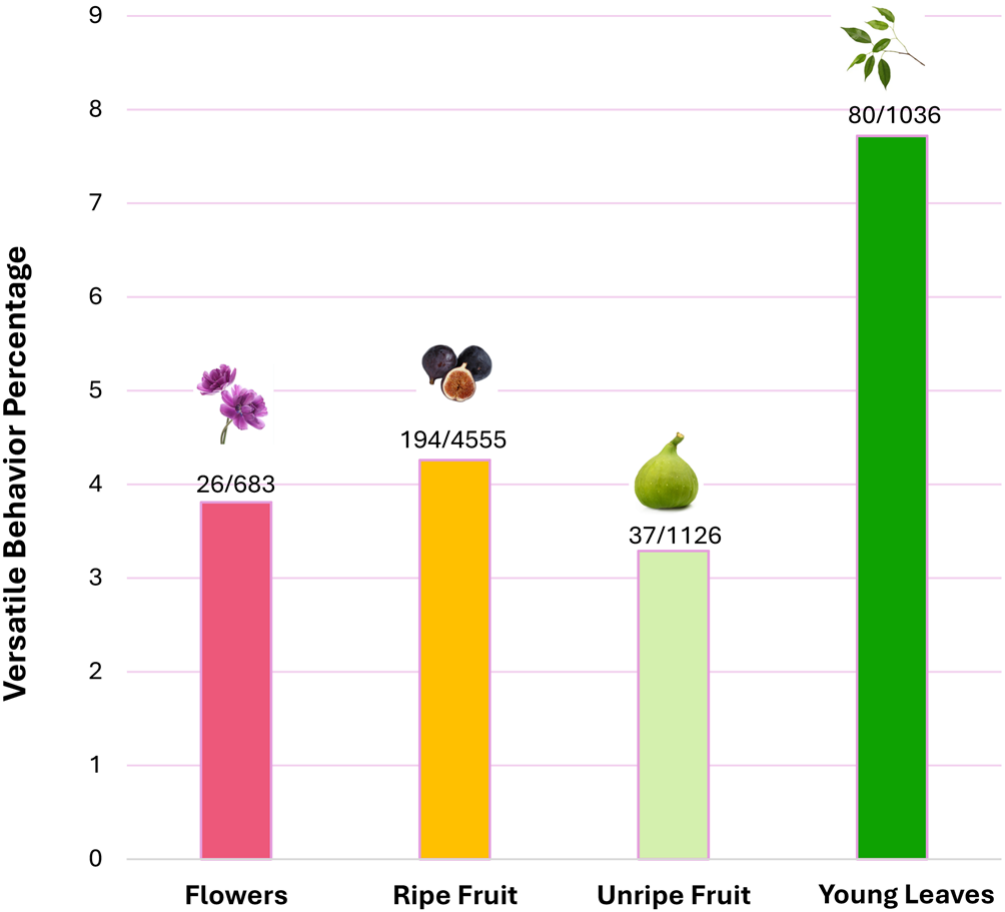
Arboreal postural versatility by food type. Above each bar, the sample size is indicated as the number of versatile postures relative to the total number of postural observations.

The percent of versatile behaviors (suspension, bipedal stand, cling, and bridge) by food category was comparable for ripe fruit (4.26%), unripe fruit (3.29%), and flowers (3.81%), but versatility while consuming leaves was considerably higher at 7.72% (Figure 2).

The size and number of supports used when feeding showed several trends. First, single supports were used much more often than multiple supports: in 83.36% of cases for leaves, 86.25% for ripe fruit, 89.51% for unripe fruit, and 81.75% for flowers.

**FIGURE 3:**
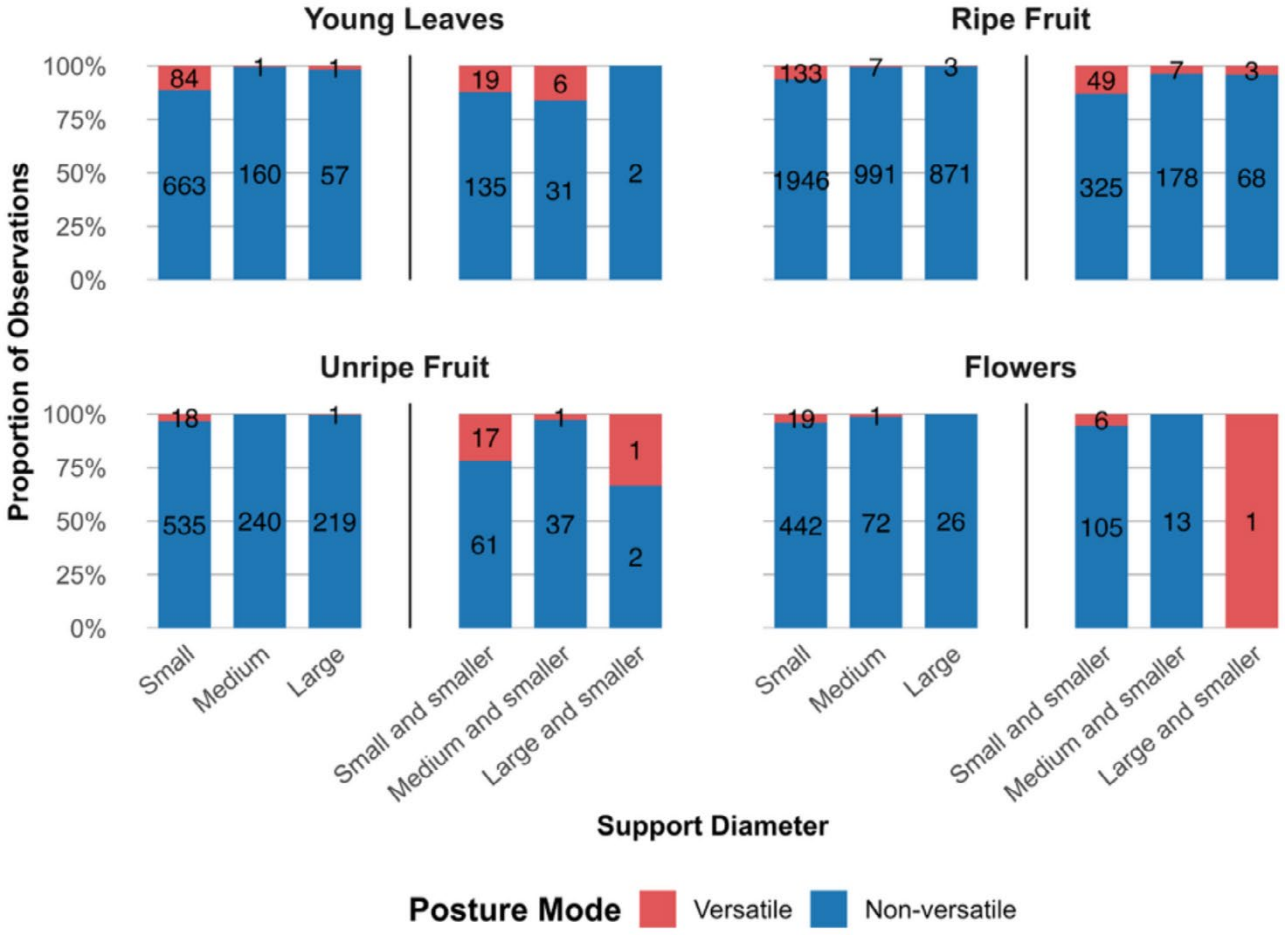
Percentage of time spent in versatile (red) versus non‐versatile (blue) postures for each single support size category (small, medium and large) and each multiple support category (small and smaller, medium and smaller, large and smaller) by food type. Definitions for support categories are in methods. Raw counts for versatile versus non‐versatile behaviors are indicated within each bar.

Second, when feeding on a single support, small‐sized supports (defined as any that could be fully encompassed by an adult hand) were generally preferred (Figure 3). However, in the case of versatile postures, this trend was magnified. For example, feeding while using a single small support (vs. any single medium or large support) accounted for 97.67% of versatile postures while consuming young leaves. This proportion was consistently high for all food types: 93.01% for ripe fruit, 94.74% for unripe fruit, and 95.00% for flowers. However, as was the case when considering versatility overall, versatility during use of single small supports was highest for leaves (11.24%), and proportions for ripe fruit (6.40%), flowers (4.12%), and unripe fruit (3.25%) were lower.

Third, using two or more small supports was more associated with versatility compared to using multiple supports with at least one medium or large support (Figure 3). For all versatile feeding postures involving multiple supports, the proportion performed on multiple small supports was 76.00% for young leaves, 83.05% for ripe fruit, 89.47% for unripe fruit, and 85.71% for flowers. On “medium and smaller” supports, this percentage was 34.00% for young leaves, 11.86% for ripe fruit, 5.26% for unripe fruit, and 0.00% for flowers. For the “large and smaller” multiple support category in which at least one support could not be fully encompassed by two adult hands, the proportions were 0.00% for young leaves, 5.08% for ripe fruit, 5.26% for unripe fruit, and 14.29% for flowers.

**FIGURE 4:**
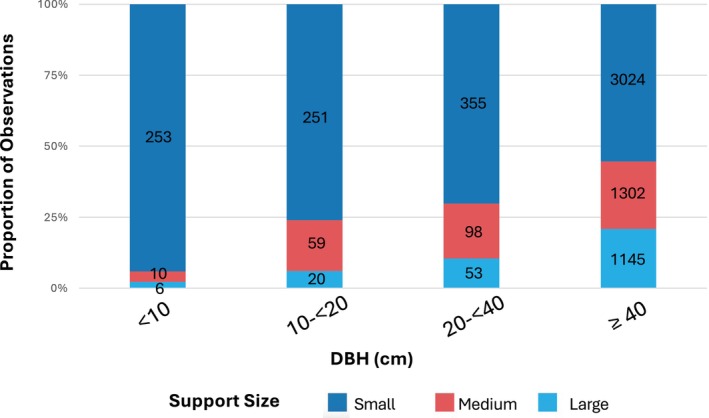
Percentage of time spent feeding on small (dark blue), medium (red) and large (light blue) supports for each diameter at breast height (DBH) category. Definitions for support diameter categories are in methods. Raw counts for each support size category are indicated within each bar.

We assessed the relationship between tree size (DBH) and support size use when only one support was used for all feeding. As noted above, chimpanzees prefer small supports overall. However, there was a consistent trend in which increasing DBH was associated with increasingly large support use (Figure 4). The percentage of large support use for trees with DBHs of < 10, 10–< 20, 20–< 40 and ≥ 40 cm were 2.23%, 6.06%, 10.47%, and 20.93%, respectively. Conversely, small‐size supports were used less and less with increasing DBH. The proportion of use from the smallest to the largest DBH category was 94.05%, 76.06%, 70.16%, and 55.27%, respectively.

**FIGURE 5:**
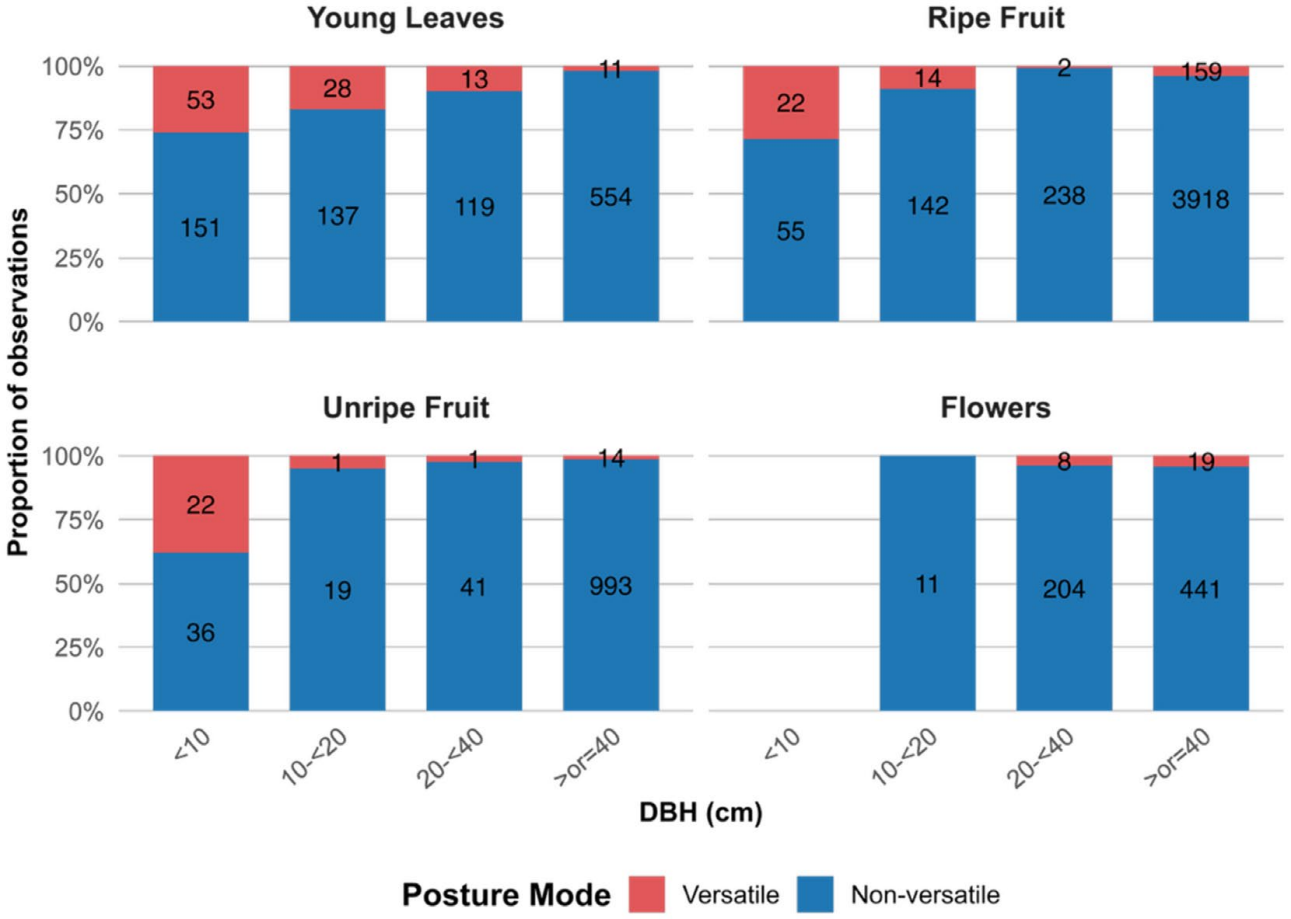
Percentage of time spent in versatile (red) versus non‐versatile (blue) postures for each diameter at breast height (DBH) category by food type. Raw counts for versatile vs. non‐versatile behaviors are indicated within each bar.

For young leaves and both categories of fruit, the proportion of versatile versus non‐versatile postures declined steeply with DBH (Figure 5). In the smallest trees with DBH < 10 cm, versatile postures for young leaves, ripe fruit and unripe fruit were 26.03%, 28.57% and 37.93% of postures, respectively, while in the largest trees with DBH ≥ 40 cm, versatility was much lower: 1.95% for young leaves, 0.38% for ripe fruit and 1.39% for unripe fruit. Flowers were not consumed in trees with DBH < 10 cm, and the pattern of versatility relative to tree DBH was different: 0.00% for DBH 10– < 20, 3.77% for DBH 20–< 40 cm and 4.13% for trees with DBH ≥ 40 cm.

Postural versatility during feeding also varied substantially when assessed across tree species while considering DBH (Table 3). Young leaf consumption was spread across multiple species. Versatility in the five most frequently consumed species was 4.84% for *M. lactea*, 0.49% for *Ficus exasperata*, 15.22% for *Pterygota mildbraedii*, 0.66% for *Ficus variifolia*, and 32.00% for *Celtis mildbraedii*. One‐hundred percent of the DBHs for *F. exasperata* and *F. variifolia* exceeded 20 cm; this percentage was lower for *M. lactea* (75.89%), while most leaf‐eating in *C. mildbraedii* and especially *P. mildbraedii* occurred in trees with DBHs < 20 cm. Thus, young leaf consumption seems clearly correlated with tree size, with high versatility only observed in smaller‐diameter species.

**TABLE 3:** Versatility by food category, species, diameter at breast height, and dietary percentage for species with > 50 observations.

Food category species^a^	Total count	Versatile count	% versatility	% DBH^b^ ≥ 20 cm	% of diet in food category^c^	% of arboreal diet^d^
**Flowers**						
*Morus lactea*	416	24	5.77	98.08	60.73	5.21
*Pterygota mildbraedii*	91	1	1.10	100.00	13.28	1.14
*Antiaris toxicaria*	63	0	0.00	100.00	9.22	0.79
**Unripe fruit**						
*Ficus mucuso*	634	4	0.63	100.00	55.91	7.94
*Pterygota mildbraedii*	53	0	0.00	100.00	4.67	0.66
*Morus lactea*	51	2	3.92	100.00	4.50	0.64
*Ficus variifolia*	50	3	6.00	100.00	4.41	0.63
*Uvariopsis congensis*	50	23	46.00	0.00	4.41	0.63
**Ripe fruit**						
*Ficus mucuso*	2450	27	1.10	97.71	53.79	30.70
*Chrysophyllum albidum*	325	2	0.62	99.69	7.14	4.07
*Morus lactea*	284	43	15.14	97.53	6.23	3.56
*Ficus dawei*	236	9	3.81	100.00	5.18	2.96
*Ficus natalensis*	191	34	17.80	100.00	4.19	2.39
*Ficus variifolia*	144	15	10.42	100.00	3.16	1.80
*Pterygota mildbraedii*	126	7	5.56	87.30	2.75	1.10
*Ficus cyathistipula*	88	8	9.09	100.00	1.92	1.10
*Cordia millenii*	90	3	3.41	97.67	1.96	1.13
*Ficus brachylepis*	80	2	2.50	100.00	1.74	1.74
*Uvariopsis congensis*	79	20	25.32	0.00	1.72	0.99
*Zanha golungensis*	78	8	10.26	98.71	1.70	0.98
*Celtis durandii*	57	2	3.51	22.81	1.24	0.71
*Ficus exasperata*	56	0	0.00	100.00	1.22	1.22
**Young leaves**						
*Morus lactea*	254	13	5.12	75.89	21.88	3.11
*Ficus exasperata*	205	1	0.49	100.00	19.32	2.57
*Pterygota mildbraedii*	199	43	21.61	5.88	17.14	2.49
*Ficus variifolia*	151	1	0.66	100.00	13.01	1.89
*Celtis mildbraedii*	52	18	34.62	34.61	4.48	0.65

^a^ All species with > 50 observations for food type in our data set.

^b^ Diameter at breast height.

^c^ Total count for tree species/total count for food category.

^d^ Total count for tree species/total count for all food (7980).

Both fruit and flower consumption tended to be dominated by a single species. Most ripe fruit was consumed in *Ficus mucuso* (53.36% of ripe fruit observations) where DBH was ≥ 20 cm for 97.71% of trees and versatile postures occurred in 1.10% of observations. For the next five most commonly consumed species (all with DBH ≥ 20 cm at rates close to or equal to 100%), versatility was high in two figs, *F. natalensis* and *F. variifolia* (17.80% and 10.42%, respectively) and in *M. lactea* (15.14%), and low in *Ficus dawei* (3.81%) and especially *Chrysophyllum albidum* (0.62%). Although ripe fruit of the understory tree *Uvariopsis congensis* was rarely consumed during the study period (1.73% of ripe fruit observations), feeding on this species was marked by high versatility (25.32%), with all observed trees having DBHs below 20 cm.

Postural versatility during unripe fruit consumption also varied widely by species. Among the large trees (DBH ≥ 20 cm), versatility was < 1% in the staple fig *Ficus mucuso* (56.31% of unripe fruit observations) and in *P. mildbraedii* trees but was higher in *M. lactea* (3.92%) and *F. variifolia* (6.00%). Versatility was highest while feeding in the understory tree *U. congensis* (DBH < 20 cm for all trees), at 46.00%, similar to the pattern observed for ripe fruit.

*Morus lactea* was the most commonly consumed flower species (60.76% of flowers consumed), with versatility comparable to the average across all food categories (5.54%). Flowers were consumed in relatively few species, and primarily in large trees with DBH ≥ 20 cm.

### 3.2 Generalized Linear Mixed Models

#### 3.2.1 GLMM Including All Trees (Model 1)

**FIGURE 6:**
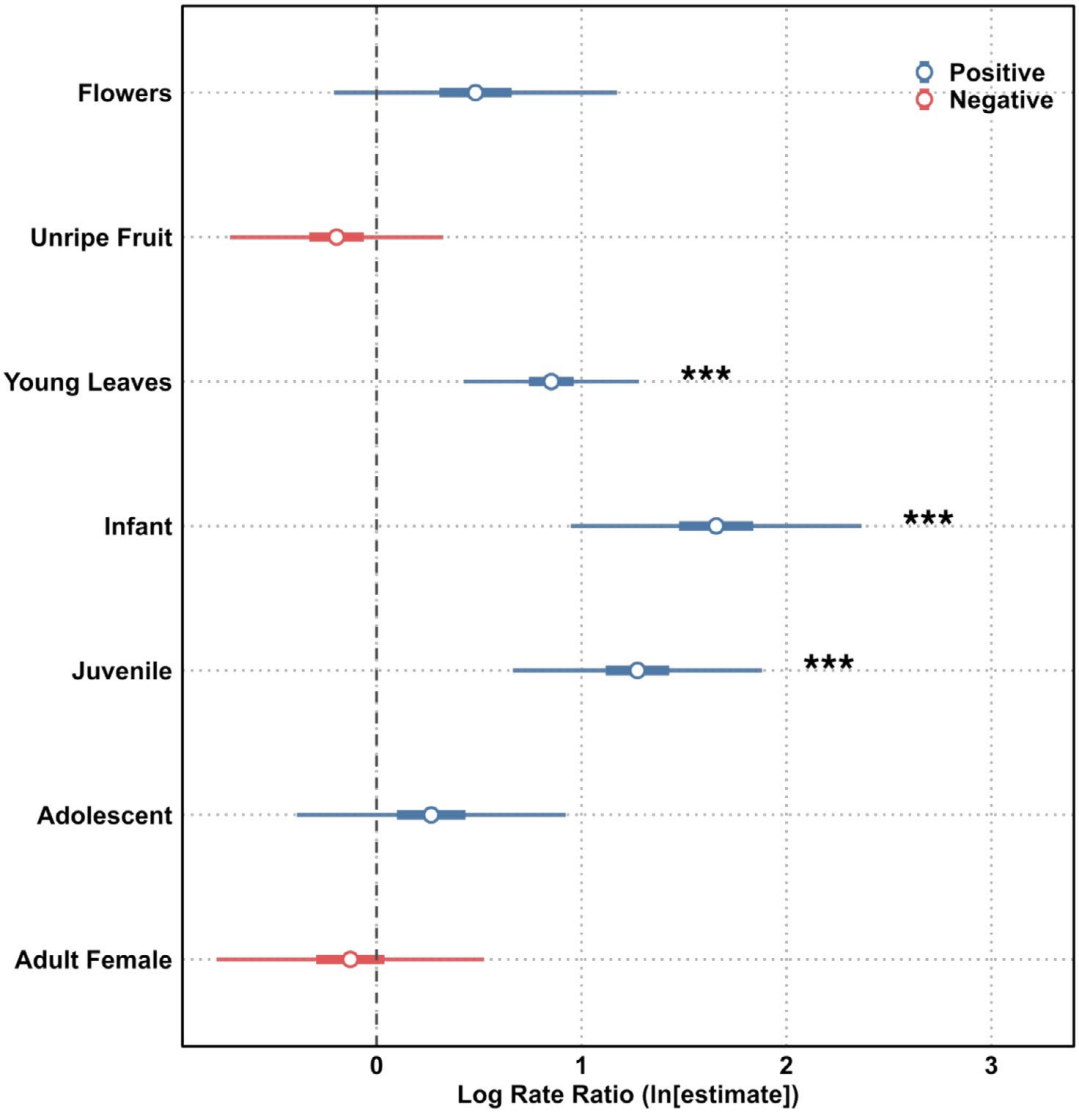
Log rate ratios predicting versatile postural behavior from the negative binomial GLMM including all trees (Model 1: Versatility ~ Food + Age Group + (1 | ID)). Log‐transformed rate ratios are shown with 95% confidence intervals (thin bars) and 50% confidence intervals (thick bars). Asterisks denote statistical significance (***p < 0.001).

Compared to eating ripe fruit (the reference category), leaf eating was associated with a 2.35‐fold increase in the rate of versatile postures (*p* < 0.001), while unripe fruit and flowers did not differ significantly from the level of versatility for ripe fruit (Figure 6, Table S2). Age group was a strong predictor of postural versatility. Infants and juveniles were 5.24 and 3.57 times as likely, respectively, to engage in versatile postures compared to adult males (the reference category; *p* < 0.001 for both). In contrast, versatility in adolescents and adult females did not differ significantly from the level in adult males.

#### 3.2.2 GLMMs for Trees With DBH ≥ 20 cm (Models 2 and 3)

**FIGURE 7:**
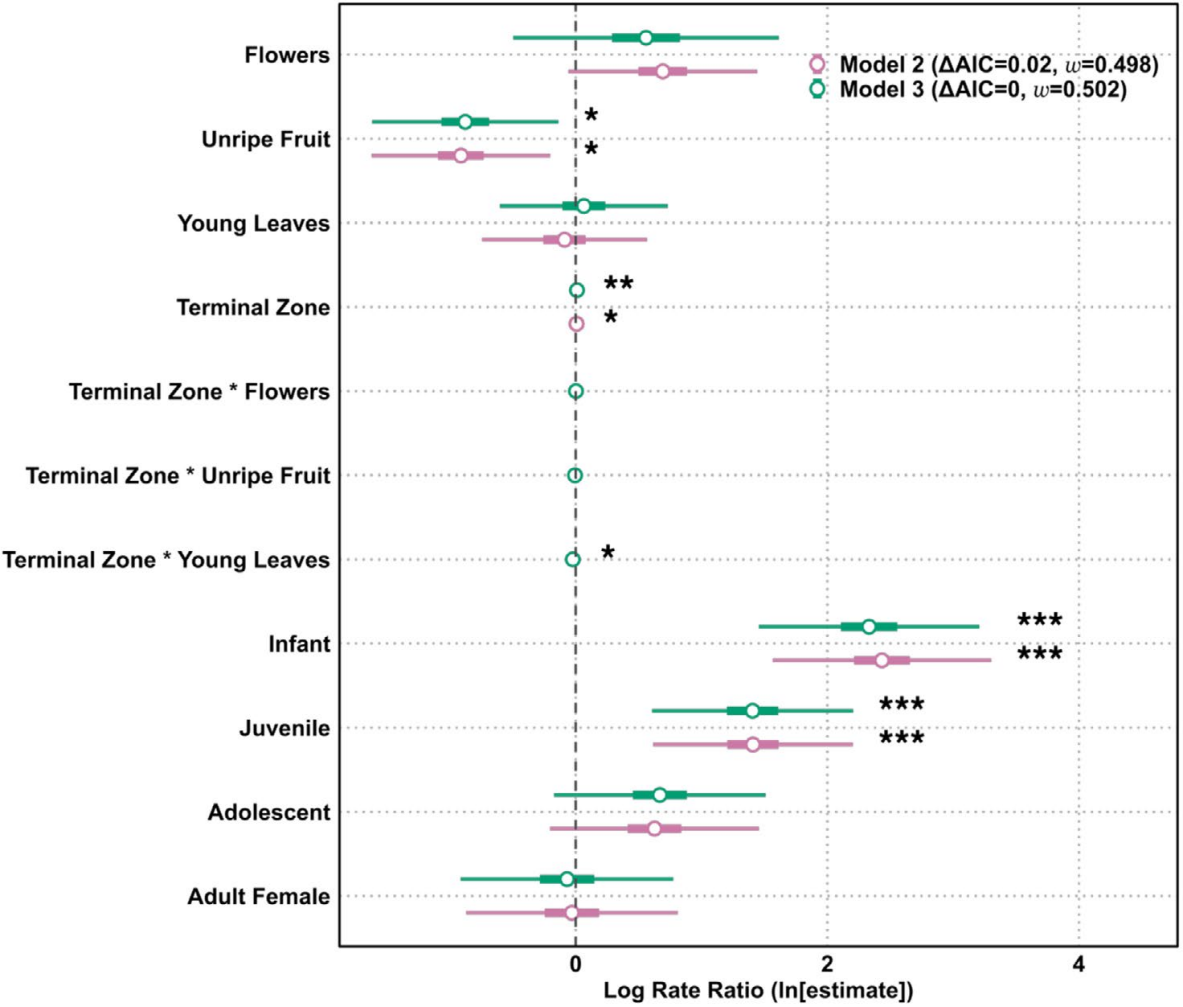
Log rate ratios predicting versatile postural behavior from the negative binomial GLMMs including only trees ≥ 20 cm DBH (Model 2: Versatility ~ Canopy zone + Food + Age Group + (1 | ID), purple; Model 3: Versatility ~ Canopy zone × Food + Age group + (1 | ID), green). Log‐transformed rate ratios are shown with 95% confidence intervals (thin bars) and 50% confidence intervals (thick bars). Asterisks denote statistical significance (*p < 0.05, **p < 0.01, ***p < 0.001).

Food type and age group were common predictors in both Model 2 and 3, and their effects on use of versatile behavior yielded almost identical statistical results (Figure 7, Tables S3 and S4). Location in the terminal as opposed to the non‐terminal zone was found to have a positive effect on versatility in both models, albeit at a higher degree of significance in Model 2 (*p* < 0.01) than Model 3 (*p* < 0.05). Below, we focus on the results of Model 3 as they allow us to examine interactions between canopy zone and food.

Age group remained a strong predictor of versatility, as in Model 1 including all trees. The expression of versatile behavior among infants was estimated to be over 10 times greater than in the adult males (*p* < 0.001), while juvenile versatility rates were approximately four times greater (*p* < 0.001). Effects for adolescents and adult females were not significant.

The results of the analysis for the effect of food type on versatility that included all trees (Model 1) differed in two major ways from the two models run on data from only big trees (Models 2 and 3). Although young leaf feeding still had a positive effect on versatile behaviors, this effect was not significant. Feeding on unripe fruit versus ripe fruit was associated with a 59.4% decrease in versatility (*p* < 0.05), while feeding on flowers rather than ripe fruit had a positive but non‐significant effect on the expression of versatile postures.

Terminal zone use had a positive impact on postural versatility although the magnitude was small, amounting to a 1.01% increase in the expected versatility. The effect of the interaction between terminal zone use and young leaves was significantly negative, indicating the positive association between versatility and terminal zone was reversed when chimpanzees were feeding on young leaves (−2.3% decrease, *p* = < 0.05) as opposed to ripe fruit. No significant interactions were observed between terminal zone use and either unripe fruit or flower consumption.

**FIGURE 8:**
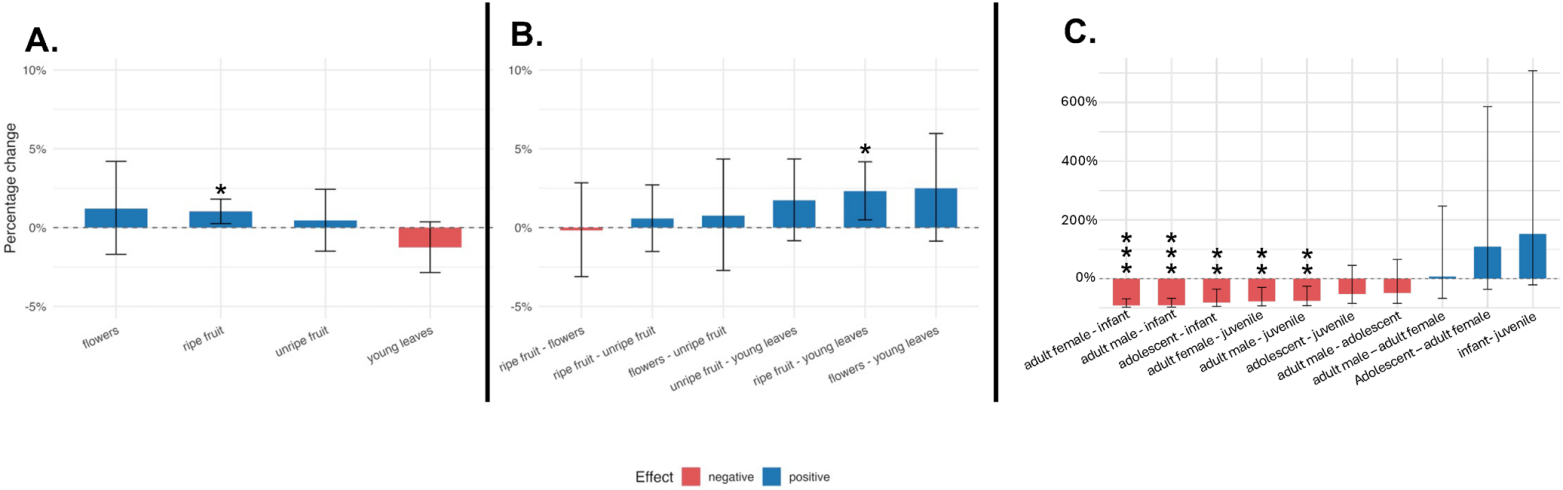
Results from a negative binomial GLMM including only trees ≥ 20 cm DBH (Model 3: Versatility ~ Canopy zone × Food + Age Group + (1 | ID)) using the emmeans package in R, including the emtrends() function. (A) Estimated slopes showing the effect of terminal zone use on foraging versatility for each food type. Slopes are exponentiated to reflect percent change in expected versatility per unit increase in terminal zone occupancy. (B) Pairwise contrasts of the slopes, expressed as exponentiated percent differences. (C) Pairwise contrasts between age groups, expressed as exponentiated percent differences. For all plots, bars represent 95% confidence intervals. Asterisks denote statistical significance (*p < 0.05, **p < 0.01, ***p < 0.001). Blue bars indicate positive effects, and red bars indicate negative effects.

We further investigated the impact of food type on versatility using the emtrends function to estimate the slope of terminal zone separately for each food category (Figure 8A). Terminal zone use was significantly associated with increased versatility when feeding on ripe fruit (*p* < 0.01), but not for other food types. In pairwise comparisons (Figure 8B), the slope for leaves was significantly lower than that for ripe fruit (2.31% increase, *p* < 0.05). All other contrasts were not significant.

Pairwise comparisons of age groups using the emtrends function reinforced the finding of substantial age‐related differences in versatility (Figure 8C). Versatility was significantly higher in infants than juveniles, and both of these age groups had higher versatility than adults. Infants had higher versatility than adolescents, but juveniles and adolescents did not differ significantly from one another, nor did adolescents and adults.

## 4 Discussion

### 4.1 Fruit, Leaves and the Terminal Canopy Zone

The chimpanzees at Ngogo, like all chimpanzees, predominantly eat ripe fruit, and like other catarrhines, mostly sit while feeding in the trees (Nowak and Reichard 2016a). Versatile feeding postures are rare events in a chimpanzee's day and are used only 5.25% of the time.

Rare events have been shown to be adaptively significant. Research on great apes and other primates helped shape the hypothesis that fallback foods, eaten only when preferred foods are unavailable, play a crucial role in defining an organism's ecological niche (e.g., Wrangham et al. 1998; Lambert et al. 2004; Marshall and Wrangham 2007; Marshall et al. 2009). The study of rare positional behaviors, particularly in chimpanzees, has also occupied a prominent place in anthropological research. For example, studies have found bipedal locomotion accounts for only 0.4% to 1.8% of adult locomotor behavior in chimpanzees, while bipedal standing makes up just 0.4% to 1.7% of adult postural behaviors (Hunt 1991; Sarringhaus et al. 2014). Despite their infrequency, the occurrence and context of such behaviors are considered key for understanding the evolution of bipedalism in hominins (Hunt 1996; Stanford 2006; Drummond‐Clarke et al. 2022; Sarringhaus et al. 2024). Hunt (1992a, 1992b, 2016) has emphasized the value of studying rare behaviors for understanding what makes chimpanzees and other apes adaptively distinct. He noted that while forelimb hanging accounts for a very small percentage of arboreal postures in chimpanzees, it occurs more frequently than in many well‐studied cercopithecoids where it is almost never observed (Hunt 2016).

Notably, infrequent versatile behaviors arise from a suite of derived anatomical features that have fundamentally shaped the niche of chimpanzees and other great apes. By examining the ecological context under which these rare behaviors occur, we now have a clearer understanding of their adaptive significance.

Our study provides support for a *terminal zone effect*. Although the magnitude of this effect is small, corresponding to a 1.0% increase in expected versatility for each percent increase in terminal zone use, it indicates that the chimpanzees at Ngogo are more likely to engage in versatile postures, particularly torso orthograde forelimb suspension, when they are feeding in the terminal rather than in the non‐terminal canopy zone. However, the impact of feeding in the terminal zone varies depending on the food being consumed. In our investigation of interactions between canopy zone and food type, we found that while eating ripe fruit, terminal zone use was significantly associated with increased versatility (8a). This finding matches a key aspect of previous terminal canopy‐frugivory frameworks (see Hunt 1992a) that predict terminal branch feeding increases the likelihood of versatility, and that this trend will be evident when consuming ripe fruit. It is noteworthy that this effect emerged despite considerable variability in the versatility associated with feeding among the large tree species that were major fruit sources during our study period (Table 3).

When feeding on young leaves in large trees, use of the terminal zone is associated with a negative effect on versatility relative to fruit eating (Figures 7 and 8B). As with the effect of feeding in the terminal zone, this relationship is modest (2.3% decrease in expected versatility for each percent increase in terminal zone use) but statistically significant.

But within the Ngogo forest overall, chimpanzees are more than twice as likely to engage in versatile postures when feeding on young leaves as opposed to ripe fruit. This is a surprising finding not predicted by hypotheses linking versatility and the origin of its anatomical underpinnings, specifically with feeding arboreally on ripe fruit. This strong, positive effect evaporates when only large trees are included in our models, but even so, when only considering large trees, the likelihood of engaging in versatile postures when feeding in the canopy on fruit is not higher than when feeding on leaves. Given that the proportion of versatile feeding postures tends to decrease with increasing tree size, the absence of a fruit effect (based on an assumption that frugivory inherently promotes versatility) may have been influenced by leaf‐eating in relatively small‐sized trees of two species, *P. mildbraedii* and *C. mildbraedii*, which together accounted for 21.62% of leaf consumption and were both associated with high versatility. *Pterygota* leaves are the most consumed leaves in the Ngogo chimpanzee diet and serve as a fallback food when non‐fig fruit are less available (Watts et al. 2012b). Most *Pterygota* and *C. mildbraedii* were excluded from the GLMMs restricted to trees ≥ 20 cm DBH, and in these models (Models 2 and 3), no significant difference in versatility was observed during the consumption of young leaves compared to ripe fruit. Another factor that may moderate versatility while leaf‐eating in large trees compared to small trees is the occurrence of tree‐wide leaf flushes. In conditions where young leaves are distributed throughout the tree, chimpanzees may prefer to sit on larger, more stable supports, a pattern observed among siamangs (Chivers 1974).

We also investigated the relationship between versatility and two other food types that are rarely considered in positional behavioral ecological hypotheses: flowers and unripe fruit. In the model that included all trees (Model 1), neither flower nor unripe fruit consumption impacted versatility relative to ripe fruit, while in our models limited to trees with DBH ≥ 20 cm, unripe fruit consumption was associated with decreased versatility relative to ripe fruit. However, *while* feeding in the terminal zone, there were no significant effects on postural versatility while eating either flowers or unripe fruit compared to when eating ripe fruit.

We had no specific hypotheses associated with the consumption of unripe fruit and flowers, and the results of our analyses are ambiguous. For the most part, eating flowers or unripe fruit exhibited few statistically significant differences from feeding on ripe fruit. We also failed to detect clear differences between eating flowers or unripe fruit versus young leaves. In our investigation of the effect of canopy position on versatility across food types, we found no significant difference between flowers and young leaves, or between unripe fruit and young leaves (Figure 8A,B). In the case of flowers, our inability to detect clear effects may be attributable, at least in part, to a smaller sample size and wider confidence intervals. Given how equivocal these findings are, further investigation is needed on how these food sources, and their position in the tree crown, impact positional behavior.

### 4.2 Tree Size, Support Size, and Support Number

Versatile behaviors are associated not only with small trees but with specific arboreal architectures, as expected. The prediction that greater versatility would be found in smaller trees and single, smaller supports was confirmed. Likewise, distributing weight across multiple supports was found to promote versatility when those supports were smaller.

Although not part of our predictions, we found that single small supports were preferred across food types and for both non‐versatile and versatile behaviors, although the use of small‐sized supports was more common during versatile behaviors. The preference for small supports while feeding in general may reflect several factors. First, over 70% of all feeding occurred either on small‐sized trees (9.6%) where small supports predominated or in the terminal canopy zone of large trees (61.0%) where support sizes are expected to be smaller than in the non‐terminal zone. Second, we found that small supports in large trees appeared to be quite stiff and therefore still suitable for sitting, as we observed minimal displacement and rebound when chimpanzees sat on and then left these supports. Future research in great ape positional behavioral ecology would benefit from a consideration of the combined role of support compliance, tree size, support size and support number in shaping behavioral patterns. Third, even while sitting, we observed that chimpanzees often lightly grasp other supports for balance with the hands or feet. We did not consider a support to be weight bearing unless we could infer that more than the weight of the limb in contact with the support was born on that support (see Hunt et al. 1996). Thus, use of the hands and feet for “balance” likely plays a major role in enabling the exploitation of small supports, including during sitting, although we did not assess it. Finally, we note that in the case of the preference for using small supports during versatile postures in particular, hand size may play a role, as it may be less feasible to suspend from larger rather than smaller supports. However, Hunt (1991) found that while chimpanzees use hook grips most commonly on substrates 2.5 cm in diameter (our small category) while suspending, they were also documented suspending on substrates up to 40.6 cm in diameter (our medium or even large size category). While this demonstrates a range of abilities, further information on substrate compliance and availability is needed to assess support preference during suspension.

### 4.3 Age

As predicted, increasing age had a negative effect on the likelihood of versatility. All age‐sex patterns match earlier findings in positional behavioral ontogeny among the Ngogo chimpanzees (Sarringhaus et al. 2014) with infants exhibiting the most versatility, followed by juveniles and then equally versatile adolescents and adults (of both sexes). Once achieving adolescence, chimpanzees are active across a range of body sizes from ~30–50 kg (Uehara and Nishida 1987; Pusey et al. 2005), and variation within this range appears to have minimal impact on the frequency of rare, versatile behaviors.

### 4.4 Beyond the Terminal Canopy Frugivory Hypotheses

Amid these broad patterns, we also ask how our findings may be enriched by considering species composition and phenology at the time of our study. As noted above, variation in the characteristics and availability of the sources of young leaves warrants further investigation and the same is true for fruit species. For example, although versatility during ripe fruit consumption was relatively high in some large trees, it was highest in small *Uvariopsis* trees (25.32% versatility overall), where the sample size was unfortunately small, just 99 observations.

If combined with the unripe fruit count (49 observations, 44.90% versatility overall), *Uvariopsis* comprises just 1.62% of arboreal feeding time. This is notable, because in an eight‐year study of the diet of the Ngogo chimpanzees, *Uvariopsis* comprised 9.98% of the total feeding time (Watts et al. 2012a), more than six times the proportion of arboreal feeding time in our study. *Uvariopsis* exhibits synchronous fruiting within local patches (Janmaat et al. 2012) and typically fruits during the rainy seasons (Kagoro‐Rugunda and Hashimoto 2015). At Ngogo, Watts et al. (2012b) found *Uvariopsis* to be fruiting somewhere in the chimpanzees' range about one‐third of the time. However, the interval between territory‐wide fruit availability was as long as 17 months (*ibid*.). Had *Uvariopsis* been more common during our 10‐month study period, it may have increased the overall percentage of versatility associated with frugivory, depending on other fruit sources. However, under such hypothetical circumstances, a higher availability of *Uvariopsis* fruit, which is found only in smaller trees, would likely have affected our findings related to versatility across all substrates, but not when considering only those trees with ≥ 20 cm DBH (i.e., with a terminal zone). During our study period, as in the eight year investigation mentioned above (Watts et al. 2012a), *Ficus mucuso* was the dominant fruit species (38.64% of arboreal feeding time, including both ripe and unripe fruit). Trees were almost all ≥ 20 cm DBH and versatility was very low (1.10% for ripe fruit and 0.63% for unripe fruit).

Tree fruiting in tropical forests is unpredictable across both annual (seasonal) and interannual scales (e.g., Chapman et al. 1997; Polansky and Boesch 2013), as illustrated by *Uvariopsis* productivity at Ngogo. It is also tremendously variable spatially, even at the local level. The Ngogo chimpanzees live ~10 km away from chimpanzees at another long‐term study site, Kanyawara. Despite this short distance, variability in food availability between these sites is pronounced (Chapman et al. 1997; Potts et al. 2011). In one 19‐month study, only eight of the top 20 plant species and just three of the top 10 overlapped between the Ngogo and Kanyawara chimpanzees (Potts et al. 2011).

While informative, these results derive from two chimpanzee communities inhabiting an old‐growth forest in Uganda. Across equatorial Africa, there is regional variation in chimpanzee habitats in terms of altitude, climate, and degree of anthropogenic impact. Populations live in arboreal environments that range from moist evergreen forest (Tai Forest, Ivory Coast; Boesch and Boesch‐Achermann 2000) to semi‐deciduous tropical forest (Gombe, Tanzania; Collins and McGrew 1988) to relatively open savanna woodland mosaics (e.g., Fongoli, Senegal; Pruetz 2006).

Nonetheless, this study of forest‐dwelling chimpanzees introduces a framework for generating hypotheses about how spatial and temporal variation in food availability—across both local and regional contexts and over annual and interannual timescales—may have shaped the evolution of positional behavior in great apes.

Our findings enable us to extrapolate beyond forested environments, as we provide evidence that diverse ecological contexts, and thus multiple selective pathways, can favor the evolution of great ape positional versatility. Food type and variation in its availability may, in different combinations, lead to equifinality in positional behavior; that is, different ecological scenarios can result in similar behavioral outcomes.

For example, we found that feeding in the terminal zone (rather than more centrally) in large‐diameter trees is associated with versatility, and that this effect holds for fruit, but not for leaves. However, we also observed that consuming young leaves in smaller trees is associated with the highest levels of postural versatility overall. Thus, both ripe fruit and young leaf consumption appear to promote versatility, with their influence shaped by multiple factors.

Variability in vegetational physiognomy and seasonality patterns provides further insights into selective pressures related to versatility. Since small trees and small supports are associated with versatility, environments in which the proportion of small trees is higher, such as woodlands compared to forests, could favor the evolution of positional versatility. Longer dry spells in woodlands, reflecting increased seasonality compared to forests, may lead to greater dependence on fallback foods such as leaves. Additionally, temporal unpredictability in fruiting patterns, since many tropical trees do not fruit on a regular annual cycle, further affects resource availability and therefore may influence versatile behavior engagement. Collectively, our data underscore a wider array of ecological drivers shaping great ape arboreal positional feeding behavior than previously recognized.

Assuming the widely accepted premise that anatomical traits associated with orthogrady and limb mobility contribute to the versatility of great ape positional behavior, this investigation provides additional context for understanding the potential selective pressures influencing the evolution of these anatomical features.

In the case of the early Miocene ape *Morotopithecus*, it becomes less surprising that foraging in seasonally dry woodlands with relatively smaller trees could have selected for the vertebral traits associated with orthogrady (e.g., a more dorsally positioned origin of the transverse processes) that would have facilitated more independent use of the limbs. This increased potential for positional versatility would have enabled these apes to better exploit woodland arboreal resources, even at large body size. Similarly, periods of fruit scarcity may have increased reliance on young leaves found on saplings, again favoring the evolution of postcranial traits linked to versatility, as well as dental traits (e.g., well‐developed molar shearing crests) associated with folivory. These ecological conditions, for which there is abundant paleoenvironmental evidence (MacLatchy et al. 2023), and the selective pressures they imposed, may thus have contributed to the distinctive combination of anatomical features related to versatility expressed in *Morotopithecus*.

The present study suggests that diverse combinations of ecological challenges may give rise to similar adaptive solutions that can enable large‐bodied apes to thrive across a range of arboreal contexts. The ability of chimpanzees, and by extension, other apes, to feed on both fruit and leaves and exploit them in the terminal canopy zone of large trees, as well as in the crowns of smaller trees, is inextricably linked to the morphofunctional features that confer positional behavioral versatility to hominoids.

## Author Contributions

**Laura MacLatchy:** conceptualization, investigation, funding acquisition, writing – original draft, methodology, writing – review and editing, formal analysis, project administration, supervision, resources, visualization, data curation. **Sharifah Namaganda:** investigation, writing – original draft, methodology, visualization, writing – review and editing, formal analysis, data curation. **Lauren Sarringhaus:** conceptualization, investigation, funding acquisition, writing – original draft, methodology, visualization, writing – review and editing, formal analysis, project administration, supervision.

## Funding

This work was supported by the Leakey Foundation; University of Michigan; National Science Foundation, 1850328.

## Supporting information


**Table S1:** Postural category definitions.

**Table S2:** Fixed effect estimates predicting versatile postural behavior from Model 1 (Versatility ~ Food + Age Group + (1 | ID)) including all trees.

**Table S3:** Fixed effect estimates predicting versatile postural behavior from Model 2 (Versatility ~ Terminal zone + Food + Age Group + (1 | ID)) including trees with DBH ≥ 20 cm.

**Table S4:** Fixed effect estimates predicting versatile postural behavior from Model 3 (Versatility ~ Terminal zone × Food + Age group + (1 | ID)) including trees with DBH ≥ 20 cm.

## Data Availability

Data that support the findings of this study are available from the corresponding author upon reasonable request.
