# Supplementary material for: Feeding in Forest Chimpanzees: Do Food Type and Canopy Location Predict Positional Behavior?
Source: Am J Biol Anthropol. 2026 Feb 22;189(2):e70204. doi: 10.1002/ajpa.70204 (PMC12926289; doi:10.1002/ajpa.70204)
Supplement: Supplementary file 1 — Table S1: Postural category definitions. [file AJPA-189-e70204-s004.docx]

**Supplemental Table S1: Postural Category Definitions**

**Versatile Modes**

cling - **“**Flexed limb posture most common on vertical-subvertical supports” (Hunt et al. 1996: 369 P3). The torso is near orthograde with the exception of infants clinging to mothers (Sarringhaus et al. 2014 S).

orthograde stand - More than half the body weight is supported by the hindlimbs in compression with the torso orthograde (based off of P5 Hunt et al. 1996), no significant contribution from the forelimbs, torso angles approx. 45 – 90 degrees, and the hip above the knees (Sarringhaus et al. 2024 pg 3).

postural bridge - “The feet grasp a support on one side of a gap, the hands grasp support on the other side, with the body spanning the gap, in tension” (Hunt et al. 1996: 375 P14).

suspension – This category encompasses multiple modes where weight is primarily borne by one or more limb(s) suspended below the substrate.

forelimb hindlimb suspend - “Suspension by a forelimb and a foot with the trunk in a subhorizontal orientation. Limbs are typically extended.

Differs from forelimb-suspend in the more pronograde orientation of the torso, and in that the forelimb need not be completely abducted” (Hunt et al. 1996: 373 P9).

hindlimb suspend - “Suspension from the foot/feet. Differs from forelimb suspend and forelimb-hindlimb suspend in lacking support from the forelimb” (Hunt et al. 1996: 373 P12).

orthograde forelimb suspend - “[M]ore than half of the body weight is borne by the forelimb(s) grasping a support above the animal’s center of mass” (Hunt et al. 1996: 372 P8), the torso is orthograde (Sarringhaus et al. 2014 S).

orthograde quadrumanous suspend - **“**Orthograde suspend where body mass may be supported by one or both hindlimbs in equal or greater proportion than one or both forelimbs” (Thorpe and Crompton 2006: 397).

pronograde suspend - Suspension with the torso pronograde (Sarringhaus et al. 2014 S) this includes quadrumanous pronograde

suspend (Hunt et al. 1996; 373 P10) and forelimb suspend with prograde compression (Thorpe and Crompton 2006: 398).

**Nonversatile Modes**

lie - “Torso [pronograde] posture on a relatively horizontal supporting stratum, body weight borne principally by the torso. When an individual grasps a support, the extremity bears little more than its own weight. When lying on a side an individual may support the upper body with an elbow” (Hunt et al. 1996: 373 P13).

pronograde stand - Three or four limbs bear the majority of the body weight with the torso pronograde on a horizontal or near horizontal substrate (based off of P4 Hunt et al. 1996). Limbs may be extended or flexed (Sarringhaus et al. 2014 S)

sit - The ischia bear substantial portion (usually more than half) of the bodyweight; the torso is relatively orthograde (Hunt et al. 1996: 367 P1).

squat - “The body weight is borne solely by the feet/foot, both hip and knee are strongly flexed. Neither forelimbs nor Ischia bear substantial body weight. The trunk is orthograde or suborthograde and the back is typically flexed. The animal is often facing at right angle to the length of the support” (Hunt et al. 1996: 369 P2).
