# Supplementary material for: Feeding in Forest Chimpanzees: Do Food Type and Canopy Location Predict Positional Behavior?
Source: Am J Biol Anthropol. 2026 Feb 22;189(2):e70204. doi: 10.1002/ajpa.70204 (PMC12926289; doi:10.1002/ajpa.70204)
Supplement: Supplementary file 2 — Table S2: Fixed effect estimates predicting versatile postural behavior from Model 1 (Versatility ~ Food + Age Group + (1 | ID)) including all trees. [file AJPA-189-e70204-s001.docx]

**Supplemental Table S2: Fixed Effect Estimates Predicting Versatile Postural Behavior from Model 1 (Versatility ~ Food + Age Group + (1 | ID)) Including All Trees.**

| **Predictor** | **Estimate (log scale)** | **Std. Error** | **95% CI (Lower) (log scale)** | **95% CI (Upper) (log scale)** | **Z-Value** | **P-Value** | **Exp(β)** | **Percent Change** |
| --- | --- | --- | --- | --- | --- | --- | --- | --- |
| **Intercept** | -3.894 | 0.253 | -4.389 | -3.398 | -15.394 | <0.001 | 0.020 | -97.963 |
| **Flowers** | 0.483 | 0.352 | -0.207 | 1.173 | 1.371 | 0.170 | 1.621 | 62.050 |
| **Leaves** | 0.853 | 0.218 | 0.425 | 1.280 | 3.908 | <0.001 | 2.346 | 134.594 |
| **Unripe Fruit** | -0.195 | 0.265 | -0.715 | 0.325 | -0.736 | 0.462 | 0.823 | -17.742 |
| **Infant** | 1.657 | 0.362 | 0.949 | 2.366 | 4.583 | <0.001 | 5.245 | 424.466 |
| **Juvenile** | 1.273 | 0.310 | 0.666 | 1.880 | 4.110 | <0.001 | 3.571 | 257.124 |
| **Adolescent** | 0.267 | 0.335 | -0.389 | 0.922 | 0.797 | 0.426 | 1.305 | 30.549 |
| **Adult Female** | -0.128 | 0.333 | -0.780 | 0.524 | -0.385 | 0.700 | 0.880 | -12.027 |

# Estimates are presented on the log scale, with corresponding 95% confidence intervals, z-values, and p-values. Exponentiated estimates (exp(β)) are also reported, with percent change reflecting the multiplicative change in expected versatility.

# Asterisks denote statistical significance (**p* < 0.05, ***p* < 0.01, ****p* < 0.001).

# Food reference category is ripe fruit.

# Age reference category is adult male.
