# Supplementary material for: Feeding in Forest Chimpanzees: Do Food Type and Canopy Location Predict Positional Behavior?
Source: Am J Biol Anthropol. 2026 Feb 22;189(2):e70204. doi: 10.1002/ajpa.70204 (PMC12926289; doi:10.1002/ajpa.70204)
Supplement: Supplementary file 3 — Table S3: Fixed effect estimates predicting versatile postural behavior from Model 2 (Versatility ~ Terminal zone + Food + Age Group + (1 | ID)) including trees with DBH ≥ 20 cm. [file AJPA-189-e70204-s003.docx]

**Supplemental Table S3: Fixed Effect Estimates Predicting Versatile Postural Behavior from Model 2 (Versatility ~ Terminal zone + Food + Age Group + (1 | ID)) Including Trees with DBH ≥ 20cm**

| **Predictor** | **Estimate (log scale)** | **Std. Error** | **95% CI (Lower) (log scale)** | **95% CI (Upper) (log scale)** | **Z-Value** | **P-Value** | **Exp(β)** | **Percent Change** |
| --- | --- | --- | --- | --- | --- | --- | --- | --- |
| **Intercept** | -4.449 | 0.313 | -5.063 | -3.835 | -14.200 | <0.001*** | 0.012 | -98.830 |
| **Terminal Zone** | 0.007 | 0.003 | 0.000 | 0.013 | 2.054 | 0.040* | 1.007 | 0.674 |
| **Flowers** | 0.692 | 0.383 | -0.058 | 1.442 | 1.808 | 0.071 | 1.998 | 99.748 |
| **Unripe Fruit** | -0.912 | 0.362 | -1.621 | -0.202 | -2.519 | 0.012* | 0.402 | -59.823 |
| **Young Leaves** | -0.089 | 0.334 | -0.744 | 0.567 | -0.266 | 0.790 | 0.915 | -8.505 |
| **Infant** | 2.434 | 0.443 | 1.566 | 3.302 | 5.494 | <0.001*** | 11.404 | 1040.418 |
| **Juvenile** | 1.409 | 0.405 | 0.615 | 2.203 | 3.477 | <0.001*** | 4.090 | 309.042 |
| **Adolescent** | 0.626 | 0.424 | -0.204 | 1.457 | 1.479 | 0.139 | 1.871 | 87.082 |
| **Adult Female** | -0.030 | 0.429 | -0.871 | 0.811 | -0.071 | 0.944 | 0.970 | -2.986 |

Estimates are presented on the log scale, with corresponding 95% confidence intervals, z-values, and p-values. Exponentiated estimates (exp(β)) are also reported, with percent change reflecting the multiplicative change in expected versatility.

Asterisks denote statistical significance (**p* < 0.05, ***p* < 0.01, ****p* < 0.001).

Food reference category is ripe fruit.

Age group reference category is adult male.
