# Supplementary material for: Feeding in Forest Chimpanzees: Do Food Type and Canopy Location Predict Positional Behavior?
Source: Am J Biol Anthropol. 2026 Feb 22;189(2):e70204. doi: 10.1002/ajpa.70204 (PMC12926289; doi:10.1002/ajpa.70204)
Supplement: Supplementary file 4 — Table S4: Fixed effect estimates predicting versatile postural behavior from Model 3 (Versatility ~ Terminal zone × Food + Age group + (1 | ID)) including trees with DBH ≥ 20 cm. [file AJPA-189-e70204-s002.docx]

**Supplemental Table S4: Fixed Effect Estimates Predicting Versatile Postural Behavior from Model 3 (Versatility ~ Terminal zone * Food + Age Group + (1 | ID)) Including Trees with DBH ≥ 20cm**

| **Predictor** | **Estimate (log scale)** | **Std. Error** | **95% CI (Lower) (log scale)** | **95% CI (Upper) (log scale)** | **Z-Value** | **P-Value** | **Exp(β)** | **Percent Change** |
| --- | --- | --- | --- | --- | --- | --- | --- | --- |
| **Intercept** | -4.463 | 0.315 | -5.082 | -3.845 | -14.147 | <2e-16 | 0.012 | -98.848 |
| **Terminal Zone** | 0.010 | 0.004 | 0.003 | 0.018 | 2.606 | 0.009** | 1.010 | 1.027 |
| **Flowers** | 0.559 | 0.539 | -0.497 | 1.615 | 1.037 | 0.300 | 1.748 | 74.822 |
| **Unripe Fruit** | -0.878 | 0.378 | -1.618 | -0.138 | -2.324 | 0.020* | 0.416 | -58.439 |
| **Young Leaves** | 0.065 | 0.340 | -0.603 | 0.732 | 0.190 | 0.849 | 1.067 | 6.675 |
| **Infant** | 2.332 | 0.447 | 1.456 | 3.208 | 5.215 | <0.001*** | 10.297 | 929.692 |
| **Juvenile** | 1.406 | 0.408 | 0.606 | 2.205 | 3.447 | <0.001*** | 4.078 | 307.822 |
| **Adolescent** | 0.668 | 0.429 | -0.172 | 1.509 | 1.559 | 0.119 | 1.951 | 95.101 |
| **Adult Female** | -0.069 | 0.431 | -0.914 | 0.776 | -0.160 | 0.873 | 0.933 | -6.673 |
| **Terminal Zone X Flowers** | 0.002 | 0.015 | -0.028 | 0.032 | 0.117 | 0.907 | 1.002 | 0.179 |
| **Terminal Zone X Unripe Fruit** | -0.006 | 0.011 | -0.027 | 0.015 | -0.533 | 0.594 | 0.994 | -0.571 |
| **Terminal Zone X Young Leaves** | -0.023 | 0.009 | -0.041 | -0.005 | -2.488 | 0.013* | 0.977 | -2.261 |

Estimates are presented on the log scale, with corresponding 95% confidence intervals, z-values, and p-values. Exponentiated estimates (exp(β)) are also reported, with percent change reflecting the multiplicative change in expected versatility.

Asterisks denote statistical significance (**p* < 0.05, ***p* < 0.01, ****p* < 0.001).

Food reference category is ripe fruit.

Age group reference category is adult male.
